# Effects of neoadjuvant chemotherapy combined with customized tumor-type total knee arthroplasty on immune function and limb function in the treatment of malignant bone tumors around the knee joint

**DOI:** 10.12669/pjms.38.6.5433

**Published:** 2022

**Authors:** Xu Zhang, Zhigang Lang, Yitian Wang

**Affiliations:** 1 Xu Zhang, Dept. of Orthopedics, Sichuan Provincial Orthopedics Hospital, 610041 Chengdu, Sichuan, China; 2 Zhigang Lang, Dept. of Orthopedics, Sichuan Provincial Orthopedics Hospital, 610041 Chengdu, Sichuan, China; 3 Yitian Wang, Department of Orthopedics, West China Hospital, 610041 Chengdu, Sichuan, China

**Keywords:** neoadjuvant chemotherapy, customized tumor-type total knee arthroplasty, malignant bone tumors around the knee joint, immune function, limb function

## Abstract

**Objectives:**

To explore the effects of neoadjuvant chemotherapy combined with customized tumor-type total knee arthroplasty (TKA) on immune function and limb function in the treatment of malignant bone tumors around the knee joint.

**Methods:**

Sixty-one patients with malignant bone tumors around the knee joint treated in Sichuan Provincial Orthopedics Hospital from February 2018 to February 2020 were retrospectively enrolled and divided into two groups according to treatment methods. The differences in immune function indexes before and after treatment were compared between the two groups. And their postoperative complications were recorded.

**Results:**

Before treatment, no significant difference can be observed in the comparison, values between the two groups (P>0.05); After treatment, the levels in Group-A were higher than before treatment (P<0.05), but the levels were not different from those before treatment (P>0.05). In contrast, the levels in Group-B were higher than before treatment and higher than that in Group-A, while the level of CD8+ was lower than before treatment and lower than that in Group-A (P<0.05). At six months postoperatively, no significant difference was observed in the excellent and good rate of limb function between the two groups (P>0.05). There were no differences in the incidence of postoperative prosthesis complications and the incidence of adverse chemotherapy reactions between the two groups (P>0.05)

**Conclusion:**

Neoadjuvant chemotherapy combined with customized tumor-type TKA for knee malignant bone tumors is an excellent safety treatment for malignant bone tumors around the knee joint, boasting a variety of benefits, such as significantly ameliorating the immune function of patients and promoting the recovery of limb function, which is worthy of clinical application.

## INTRODUCTION

Malignant bone tumors refer to malignant tumors that occur in bone and cartilage, accounting for approximately 0.2% of all malignant tumors in the body. Such tumors are commonly seen in the distal femur and proximal tibia, with their predominant sites around the knee join.^1^ Surgery is the preferred treatment for malignant bone tumors around the knee joint, and its surgical procedures include limb salvage and amputation. Amputation is dominant in the early stage of treatment, which not only affects the quality of life of patients, but also brings numerous postoperative complications to patients.^2^ In recent years, the maturity of chemotherapy has contributed to the development of limb salvage technology, with which the survival rate and limb salvage rate of patients with primary malignant bone tumor have been greatly improved.^3^ Knee prosthesis arthroplasty is a frequently used procedure for limb salvage for knee malignant bone tumors. Studies have shown that customized tumor-type artificial prostheses have positive significance for the recovery of knee joint function in patients with malignant bone tumors around the knee joint.^4^ In this study, the effects of neoadjuvant chemotherapy combined with customized TKA on immune function and limb function of patients with malignant bone tumors around the knee joint were investigated.

## METHODS

Sixty-one patients with malignant bone tumors around the knee joint treated in Sichuan Provincial Orthopedics Hospital from February 2018 to February 2020 were retrospectively enrolled and divided into two groups according to treatment methods: Group-A (28 cases) and Group-B (33 cases).

### Ethical Approval

The study was approved by the Institutional Ethics Committee of Sichuan Provincial Orthopedics Hospital on March, 2020 (No.: 2020033), and written informed consent was obtained from all participants.

### Inclusion criteria:

• Patients diagnosed by postoperative pathological biopsy;
• Newly treated patients;
• Patients with strong desire to salvage limbs;
• Patients with complete clinical data.

###  Exclusion criteria:

• Patients with severe dysfunction of heart, liver and kidney;
• Patients with distant metastases;
• Patients intolerant to chemotherapy and surgery;
• Patients with autoimmune diseases;
• Patients in pregnancy and lactation;
• Patients who do not cooperate with treatment and postoperative follow-up;
• Patients with incomplete clinical data.

There were 15 males and 13 females in Group-A, aged from 16 to 60 years old, with an average of (36.62±11.62) years old, and a body mass index (BMI) of (23.68±2.58) kg/m^2^. Tumor types: 20 cases of osteosarcoma, 8 cases of chondrosarcoma; Tumor sites: 23 cases in the distal femur, five cases in the proximal tibia. Enneking staging: 21 cases of stage IIA, seven cases of stage IIB. There were 18 males and 15 females in Group-B, aged from 18 to 61 years old, with an average of (36.64±11.70) years old, and a body mass index (BMI) of (23.77±2.58) kg/m2. Tumor types: 23 cases of osteosarcoma, 10 cases of chondrosarcoma; Tumor sites: 26 cases in the distal femur, seven cases in the proximal tibia. Enneking staging: 24 cases of stage IIA, nine cases of stage IIB. No statistically significant difference was observed in the comparison of general data between the two groups (P>0.05).

Surgery was performed by the same senior oncology surgeon in both groups. Patients in Group-A were treated with customized tumor-type TKA: CT, MRI, chest X-ray, and radionuclide whole-body bone scan were routinely performed before surgery to exclude metastatic lesions, and tumor staging was also performed. Positive and lateral x-ray of the affected knee joint were taken, and the mark amplification ratio was referenced to customize the personalized artificial knee prosthesis. All patients received a customized new pivoted tumor knee prosthesis. During the procedure, patients were intubated with an endotracheal tube and placed under general anesthesia, their affected limb was elevated, a balloon tourniquet was applied, but a blood drain was disabled.

A medial knee incision was made through the joint capsule below the vastus medialis. The skin, subcutaneous tissue, and deep fascia were sequentially incised to separate normal soft tissue from the tumor, while care should be taken to avoid nerve and blood vessel injury. According to Enneking’s principle, extensive resection of the tumor was performed, and an osteotomy was performed 3cm from the maximum infiltration of the medullary cavity indicated by preoperative MRI for pathological examination to determine the resection boundary of the tumor. There was no need for prosthesis arthroplasty of patella. The inactivated patella innervation was performed with electric knife, and the artificial prosthesis was implanted. The range of motion, tightness and force line of lower limbs were defined, and the prosthesis was fixed with bone cement. A drainage tube was routinely indwelled at the incision. Antibiotics were dropped intravenously Three to five days postoperatively to prevent infection. Drainage tube was removed 48h postoperatively, and passive activities and functional exercises were gradually started. Five days postoperatively, patients gradually got out of bed for exercise and continued to receive postoperative chemotherapy after stitches were removed from the wound (the postoperative chemotherapy drugs were the same as before, with a total of four to five cycles).

Patients in Group-B were treated with neoadjuvant chemotherapy based on the treatment of Group-A: Neoadjuvant sequential chemotherapy was performed preoperatively, with a chemotherapy regimen of intravenous infusion of doxorubicin (60 mg/m^2^, d1-d2), cisplatin (100 mg/m^2^, d1-d3), methotrexate (8-10 g/m^2^, d16), vincristine (2 mg/m^2^, d16), ifosfamide (2 g/m^2^, d21-d25) for 2-4 cycles. Artificial joint arthroplasty was performed 2 weeks after the end of the neoadjuvant chemotherapy cycle, and the surgical method was the same as that of Group-A.

### Observation Indexes

The differences of immune function indexes and limb function between the two groups before and after treatment were compared, and their postoperative complications were recorded.

### Immune function indexes

Fasting venous blood (5 mL) was collected before and six months preoperatively and centrifuged (3000 r/min, 10 minutes). Serum was separated and cryopreserved (-80^0^C). Immunoturbidimetric method was utilized to detect the levels of immunoglobulin (IgA, IgG, IgM), and the kits used were provided by Changchun Huili Biotechnology Co., LTD. CD3+, CD4+, CD8+ levels were detected by DxFLEX flow cytometry from Beckman Instruments, Inc., USA, and CD4+/CD8+ values were calculated.

### Limb function

All patients were followed up for one year until February 2021. MSTS scoring system^5^ was utilized to evaluate the limb function of the two groups at six months postoperatively and at the last follow-up: including the scores of gait, pain, support, acceptance, walking function and overall function, with 0-5 points for each item and 0-30 points for the total score. A score of <12 indicates poor, 12-17 indicates medium, 18-23 indicates good, and 24-30 indicates excellent. The excellent and good rate = number of (excellent + good)/total number of cases * 100%.

Postoperative complications include prosthesis complications (prosthesis loosening, prosthesis infection, prosthesis rupture) and adverse chemotherapy reactions (gastrointestinal discomfort, liver and kidney damage, bone marrow suppression).

### Statistical method

All data in this study were processed by SPSS 22.0 software. Enumeration data was expressed in n (%), and x2 or Fisher’s exact probability analysis test was performed for comparison between groups; Measurement data of normal distribution was expressed in (*x̅*+S), independent sample t test was performed between groups, and paired sample t test was performed before and after treatment within the group; Mann-Whitney U test was performed for ranked ordinal data. P<0.05 indicates a statistically significant difference.

## RESULTS

Before treatment, no significant difference can be observed in the comparison of IgA, IgG, IgM, CD3+, CD4+, and CD8+ levels and CD4+/CD8+ values between the two groups (P>0.05); After treatment, IgA, IgG, and IgM levels in Group-A were higher than before treatment (P<0.05), but CD3+, CD4+, CD8+ levels and CD4+/CD8+ values were not different from those before treatment (P>0.05). In contrast, IgA, IgG, IgM, CD3+, and CD4+ levels and CD4+, CD8+ valuesin Group-B were higher than before treatment and higher than that in Group-A, while CD8+ level was lower than before treatment and lower than that in Group-A (P<0.05). Table-I.

**Table I:** Comparison of immune function indexes between the two groups before and after treatment (*x̅*+S).

Group	Time	IgA (g/L)	IgG (g/L)	IgM (g/L)	CD3+ (%)	CD4+ (%)	CD8+ (%)	CD4+/CD8+
Group-A (n=28)	Before treatment	1.75±0.47	10.49±2.30	0.89±0.25	55.47±5.46	35.36±3.67	31.48±4.29	1.36±0.40
	After treatment	2.31±0.72	12.59±3.16	1.10±0.33	53.25±6.55	34.52±3.26	32.90±4.71	1.33±0.41
	t value	-3.973	-3.399	-3.973	1.831	1.592	-1.190	0.303
	P value	<0.001	0.002	<0.001	0.078	0.123	0.057	0.764
Group-B (n=33)	Before treatment	1.70±0.41*	10.51±2.35*	0.84±0.22*	54.19±5.47*	35.64±4.53*	30.27±4.25*	1.39±0.39*
	After treatment	3.42±1.04#	14.57±4.37#	1.39±0.46#	58.54±6.49#	39.49±4.45#	25.30±3.20#	1.64±0.41#
	t value	-9.970	-5.742	-7.311	-4.368	-4.751	5.612	-2.877
	P value	<0.001	<0.001	<0.001	<0.001	<0.001	<0.001	0.007

***Note***: Compared with Group-A before treatment,

* P>0.05; Compared with Group-A after treatment,

# P<0.05.

At six months postoperatively, the excellent and good rate of limb function in Group-B (93.94% vs 75.00%) was apparently superior to that of Group-A (*P*<0.05); At the last follow-up, no significant difference was observed in the excellent and good rate of limb function between the two groups (96.43% vs 100.00%) (*P*>0.05). Table-II.

**Table II:** Comparison of limb function between the two groups after treatment [n (%)].

Time	Group	Limb function				Excellent and good rate

		Excellent	Good	Medium	Poor	
6 months postoperatively	Group-A	7 (25.00)	14 (50.00)	5 (17.86)	2 (7.14)	21 (75.00)
	Group-B	11 (33.33)	20 (60.61)	2 (6.06)	0 (0.00)	31 (93.94)
	Z/x2 value	-3.782				4.320
	P value	<0.001				0.038
Last follow-up	Group-A	15 (53.57)	12 (42.86)	1 (3.57)	0 (0.00)	27 (96.43)*
	Group-B	17 (51.52)	16 (48.48)	0 (0.00)	0 (0.00)	33 (100.00)
	Z/x2 value	-6.904				1.198
	P value	0.603				0.274

***Note***: Compared with 6 months postoperatively,

* p<0.05.

There were no differences in the incidence of postoperative prosthesis complications (7.14% vs 6.06%) and the incidence of adverse chemotherapy reactions (17.86%vs9.09%) between the two groups (*P*>0.05). Table-III.

**Table III:** Comparison of postoperative complications between the two groups [n (%)].

Group	n	Prosthesis complications			Total incidence

		Prosthesis loosening	Prosthesis infection	Prosthesis fracture	
Group-A	28	1 (3.57)	0 (0.00)	1 (3.57)	2 (7.14)
Group-B	33	1 (3.03)	1 (3.03)	0 (0.00)	2 (6.06)
x2 value	-	0.014	0.863*	0.863*	0.029
P value	-	0.906	1.000	1.000	0.865

Group	n	Adverse chemotherapy reaction			Total incidence

		Gastrointestinal discomfort	Liver and kidney damage	Bone marrow suppression	

Group-A	28	3 (10.71)	1 (3.57)	1 (3.57)	5 (17.86)
Group-B	33	2 (6.06)	1 (3.03)	0 (0.00)	3 (9.09)
x2 value	-	0.436	0.014	0.863*	1.022
P value	-	0.509	0.906	1.000	0.312

***Note***:

* indicates the test result of *Fisher’s* exact probability analysis method.

## DISCUSSION

In the wake of the uninterrupted development of medical technology in recent years, the treatment of malignant bone tumors around the knee joint has been transformed from early amputation to comprehensive treatment based on limb salvage.^6^ With the advent of neoadjuvant chemotherapy, a strong guarantee is provided for the treatment of malignant bone tumors around the knee joint by limb salvage. Neoadjuvant chemotherapy boasts a variety of efficacy, which can not only eliminate potential microscopic lesions throughout the body, reduce the volume of the primary tumor and the surrounding reaction zone, increase the probability of complete resection, but also judge the chemotherapy sensitivity of patients, thus providing reference for postoperative chemotherapy.^7^-^9^ Currently, limb salvage for malignant bone tumors around the knee joint is mainly divided into biological reconstruction and mechanical reconstruction, the former refers to inactivation and replantation of tumor segment, while the latter refers to tumor-type prosthesis arthroplasty. Patients undergoing mechanical reconstruction showed stable joint structure, reduced bed time, faster recovery of limb function, and more acceptable prostheses appearance compared with those undergoing biological reconstruction.^10^,^11^ In view of this, neoadjuvant chemotherapy combined with customized tumor-type TKA has turned into the mainstream surgical method for the treatment of malignant bone tumors around the knee joint.

Previous studies have confirmed that patients with malignant bone tumors have reduced immune function, and most of them are caused by immune dysfunction.^12^ IgA, IgG, IgM and other immunoglobulins (Ig) are sensitive indexes of humoral immunity. Specifically, IgA is principally secreted by mucosal cells of nasal and lung tissues, which is the first line of defense for humoral immunity. IgG is mainly secreted by plasma cells of spleen and lymph nodes, accounting for 60%-70% of the total amount of Ig, which is an important factor to prevent infectious diseases. Both IgM and IgG are of the same origin, but IgM is the earliest humoral immune antibody and a vital marker of recent infection.^13^-^15^ T lymphocyte subsets such as CD3+, CD4+ and CD8+ reflect cellular immune sensitivity indexes of the body. Specifically, CD3+ is widely distributed in mature T cell membranes and plays a key role in rejection, tumor cell elimination, and anti-cell infection. CD4+ has the function of regulating immune response and promoting the differentiation of other immune cells, while CD8+ can inhibit the activity of other helper T cells, thereby indirectly inhibiting the differentiation of killer T cells and B cells.^16^-^18^

It was shown in a study by Wu W et al.^19^ that the decreased immune function of patients with malignant bone tumors around the knee joint was manifested as a significant decrease in IgA, IgG, IgM, CD3+, CD4+ levels and CD4+/CD8+ values, while a significant increase in CD8+ level. As indicated by the results of this study, IgA, IgG and IgM levels in both groups were significantly improved compared with before treatment, and Group-B showed a greater degree of improvement. CD3+, CD4+, CD8+ and CD4+/CD8+ values in Group-A were not significantly improved compared with before treatment, while CD3+, CD4+ levels and CD4+/CD8+ values were increased and CD8+ value was decreased in Group-B, suggesting that neoadjuvant chemotherapy combined with customized tumor-type TKA can remarkably ameliorate the immune function of patients with malignant bone tumors around the knee joint, which is consistent with the results of Kawai A et al.^20^

In principle, the tumor needs to be completely removed during treatment, which makes it difficult to retain the supporting structures around the knee joint, thus affecting the recovery of limb function after surgery^21^. With the selection of a new customized pivoted tumor knee prosthesis in this study, the above problems have been well solved. Such a prosthesis has both good rotational stability and greater mobility, which greatly reduces the incidence of postoperative prosthesis loosening. As shown in this study, the excellent and good rate of limb function in Group-B was apparently superior to that in Group-A at six months postoperatively, and there was no significant difference in the excellent and good rate of limb function between the two groups at the last follow-up, suggesting that neoadjuvant chemotherapy combined with custom-made tumor-type TKA is beneficial to promote the recovery of limb function in patients with malignant bone tumors around the knee joint, which is the same as the results of Zhang X et al.^22^.

It was also shown in the results of this study that there was no significant difference in the incidence of postoperative prosthetic complications and adverse chemotherapy reactions between the two groups, suggesting that the safety of neoadjuvant chemotherapy combined with customized tumor-type TKA in the treatment of malignant bone tumors around the knee joint is worthy of affirmation.^23^

### Limitations of the study

It include small sample size, short follow-up time. We are actively increasing the sample size and further prolonging the follow-up time. Besides, we are further detailing the study, in order to make more objective evaluations of the influence of this therapy in different pathological types and its long-term effects.

## CONCLUSION

Neoadjuvant chemotherapy combined with customized tumor-type TKA for knee malignant bone tumors is an excellent safety treatment for malignant bone tumors around the knee joint, boasting a variety of benefits, such as significantly ameliorating the immune function of patients and promoting the recovery of limb function, which is worthy of clinical application.

### Authors’ Contributions:

**XZ:** Designed this study and prepared this manuscript, and are responsible and accountable for the accuracy or integrity of the work.

**ZL:** collected and analyzed clinical data.

**YW:** Significantly revised this manuscript.
